# The Development of Reagentless Amperometric Glucose Biosensor Based on Gold Nanostructures, Prussian Blue and Glucose Oxidase

**DOI:** 10.3390/bios13100942

**Published:** 2023-10-20

**Authors:** Laura Sakalauskiene, Benediktas Brasiunas, Anton Popov, Asta Kausaite-Minkstimiene, Almira Ramanaviciene

**Affiliations:** 1 NanoTechnas—Center of Nanotechnology and Materials Science, Faculty of Chemistry and Geosciences, Vilnius University, Naugarduko St. 24, LT-03225 Vilnius, Lithuania; laura.sakalauskiene@chgf.vu.lt (L.S.); benediktas.brasiunas@chgf.vu.lt (B.B.); anton.popov@chgf.vu.lt (A.P.); 2 Department of Immunology, State Research Institute Centre for Innovative Medicine, Santariskiu St. 5, LT-08406 Vilnius, Lithuania

**Keywords:** glucose biosensor, Prussian blue, gold nanostructures, glucose oxidase, hydrogen peroxide

## Abstract

Precise blood glucose detection plays a crucial role in diagnosing and medicating diabetes, in addition to aiding diabetic patients in effectively managing their condition. In this research, a first-generation reagentless amperometric glucose biosensor was developed by combining the graphite rod (GR) electrode modification by gold nanostructures (AuNS) and Prussian blue (PB) with glucose oxidase (GOx)—an enzyme that can oxidize glucose and produce H_2_O_2_. Firstly, AuNS was electrochemically deposited on the GR electrode (AuNS/GR), and then PB was electrochemically synthesized on the AuNS/GR electrode (PB/AuNS/GR). Finally, GOx was immobilized over the PB/AuNS nanocomposite with the assistance of Nafion (Nf) (Nf-GOx/PB/AuNS/GR). An application of PB in the design of a glucose biosensor enables an easy electrochemical reduction and, thus, the determination of the H_2_O_2_ produced during the GOx-catalyzed oxidation of glucose in the sample at a low operation potential of −0.05 V vs. Ag/AgCl/KCl_3 mol L_^−1^. In addition, AuNS increased the electrochemically active surface area, improved the GOx immobilization and ensured a higher analytical signal. The developed glucose biosensor based on the Nf-GOx/PB/AuNS/GR electrode exhibited a wide linear range, from 0.025 to 1 mmol L^−1^ of glucose, with a 0.0088 mmol L^−1^ limit of detection, good repeatability and high selectivity over electroactive interfering substances. The developed biosensor is convenient for the determination of glucose in the physiological environment.

## 1. Introduction

In the 21st century, diabetes remains a long-term and complex health problem that poses significant challenges. Due to the clinical importance of measuring blood glucose levels, numerous efforts have been undertaken to develop sensitive, selective, reliable, cost-effective and convenient glucose biosensors. The various metal and carbon nanostructures, different redox mediators and conducting polymers, as well as various electrode and surface modification procedures, were applied to facilitate a timely and precise glucose determination and diabetes management [1,2,3,4,5].

The beginning of biosensors is considered to be in 1962 when Leland C. Clark and Champ Lyons proposed the first enzymatic glucose biosensor, where glucose oxidase (GOx) was coupled to an electrode for the amperometric detection of O_2_. The biosensors for glucose determination were based on monitoring either O_2_ consumption or H_2_O_2_ production [6]. Although the first biosensors were based on O_2_ determination [7,8,9], control of the H_2_O_2_ concentration was found to be more sensitive [10]. However, the amperometric detection of H_2_O_2_ requires a high anodic potential (over +0.7 V) and is affected by interfering substances, such as ascorbic and uric acids, usually present in real samples. Thus, a solution was needed to overcome this problem. Although Prussian blue (PB) or ferric ferrocyanide was first announced in 1710 [11], and its electrochemical properties have been known, the development of amperometric biosensors based on electrodes modified with PB was first announced in 1994 [12]. Thus, a first-generation amperometric glucose biosensor, based on PB electrochemically deposited on a glassy carbon (GC) electrode, was developed for the selective and sensitive determination of H_2_O_2_ by electroreduction in the presence of O_2_ at +0.18 V (vs. Ag/AgCl/KCl_1 mol L_^−1^). Moreover, such a low potential enables the avoidance of interference from electrochemically active substances present in real samples [13]. PB can be easily synthesized on the electrode surface and can replace the electron-transfer mediator present in the solution [14,15], thereby directly generating an electrochemical signal. This ensures the advancement of reagentless electrochemical glucose biosensors, where all necessary components are integrated, eliminating the need for reagent injection during the analysis process [16,17]. Unfortunately, PB-modified layers exhibit limited operational stability, particularly in neutral and alkaline conditions. The employment of polymers like Nafion (Nf) proves to be an effective solution to address this concern [4,18].

Other important requirements for the glucose biosensor are sufficient stability and a high analytical signal at low concentrations of glucose. For this purpose, gold nanoparticles of different sizes and dendritic gold nanostructures were successfully applied, and the impact of the GOx immobilization method on the analytical signal was evaluated [7]. Gold nanostructures (AuNS) are intriguing nanomaterials that have become increasingly popular in the interface fabrication of biosensors due to their low electrical resistivity and relatively wide electrochemical potential window [19], good biocompatibility [20], improved electrooxidation and fast electron transfer [21]. AuNS enhance the electrochemically active surface area, facilitate the electron transfer between the GOx redox centers and the electrode surface in the presence of a soluble redox mediator, and improve the sensitivity and stability of biosensors [22].

The main aim of this research was the development of sensitive and reagentless glucose biosensors based on the enzyme GOx immobilized on a graphite rod (GR) electrode premodified by electrochemically deposited AuNS and PB. The advantages of AuNS, which enhance the electrochemical response, and PB, which acts as an electron-transfer mediator, were combined. The optimal conditions for the electrochemical deposition of AuNS and PB and the required amount of GOx on the modified GR electrode surface were selected. The analytical characteristics of the developed glucose biosensors were assessed. The performance of the newly developed glucose biosensor was tested for the determination of glucose in human serum samples.

## 2. Materials and Methods

### 2.1. Materials and Equipment

GOx (from Aspergillus niger, Type VII, lyophilized powder, ≥100,000 units/g solid, without added oxygen) was received from Sigma-Aldrich (Gillingham, UK). Hydrogen tetrachloroaurate (III) hydrate (HAuCl_4_·3 H_2_O), Nafion and D-(+)-glucose monohydrate were obtained from Alfa Aesar (Karlsruhe, Germany). Sulfuric acid, iron chloride (FeCl_3_), hydrogen chloride, potassium chloride and hydrogen peroxide (30%) were acquired from Carl ROTH (Karlsruhe, Germany). Potassium ferricyanide (K_3_[Fe(CN)_6_]) was received from Sigma-Aldrich (Shanghai, China). Dipotassium hydrogen phosphate (K_2_HPO_4_) was obtained from Sigma-Aldrich (Steinheim, Germany), while potassium dihydrogen phosphate (KH_2_PO_4_) was obtained from Riedel-deHaën (Seelze, Germany). Ascorbic acid was obtained from Fluka (Buchs, Switzerland) and uric acid from AppliChem (Darmstadt, Germany). Graphite rods (diameter 3.0 mm, purity-99.999%) were purchased from Sigma-Aldrich (St. Louis, MO, USA). The 0.05 mol L^−1^ phosphate buffered solution (PBS) containing 0.1 mol L^−1^ KCl, pH 5.8, was used in the experiments. The glucose solution with an equilibrium ratio of α-β optical isomers was obtained by preparing it 24 h in advance before use. All solutions were prepared using deionized water that underwent purification using the Millipore S.A. (Molsheim, France) filter system.

The electrochemical measurements were carried out using potentiostat/galvanostat PalmSens4 (PalmSens BV, Houten, The Netherlands) driven by the PSTrace 5.9 software. The surface morphology of the prepared electrode was imaged by a high-resolution field emission scanning electron microscope SU-70 (Hitachi, Japan) (FE-SEM).

### 2.2. The Preparation, Modification and Characterization of the Graphite Rod Electrode

The graphite rods were subjected to mechanical polishing using fine, very fine and ultra-fine grit sandpaper. After sanding, the GR electrodes were rinsed with deionized water, followed by air drying at room temperature (20 ± 2 °C). In addition, the electrode was enclosed in a silicone tube to prevent contact between the electrode side surface and the solution within the electrochemical cell. The geometric surface area of the GR electrodes was 0.071 cm^2^. To modify the GR electrode with the AuNS, the GR electrode was immersed in the 1.0, 3.0, 6.0 or 10.0 mmol L^−1^ solution of HAuCl_4_ with 0.2 mol L^−1^ H_2_SO_4_. Electrochemical synthesis of the AuNS was performed at a constant −0.2 V potential vs. Ag/AgCl/KCl_3 mol L_^−1^ for 120 s, according to an earlier published protocol [23]. The working electrode after electrochemical AuNS synthesis was rinsed with deionized water, dried at room temperature and characterized by FE-SEM.

An aqueous solution of 0.50 mol L^−1^ H_2_SO_4_ was used to determine the electrochemically active surface area (*EASA*) of the AuNS/GR using the cyclic voltammetry (CV) method. Cyclic voltammograms were recorded in the potential range from 0.0 to +1.4 V vs. Ag/AgCl/KCl_3 mol L_^−1^ at a scan rate of 100 mV s^−1^. The *EASA* was estimated using the following Equation (1) [3]:

(1) $$EASA=\frac{A}{\nu \cdot 386\;\mu C\;cm^{-1}},$$

where *A* is the integrated peak of gold oxide reduction, *v* is the potential scan rate (V s^−1^) and 386 µC cm^−1^ is the charge density per unit area associated with the electrochemical reduction of a monolayer of chemisorbed oxygen on polycrystalline gold.

The synthesis and deposition of PB on the AuNS/GR electrode were performed electrochemically. To achieve that, the GR electrode modified with the AuNS was immersed in an aqueous solution consisting of 3.0 mmol L^−1^ K_3_[Fe(CN)_6_], 3.0 mmol L^−1^ FeCl_3_, 0.1 mol L^−1^ KCl and 0.1 mmol L^−1^ HCl, and a constant +0.4 V potential vs. Ag/AgCl/KCl_3 mol L_^−1^ for 150 s was applied to the working electrode. After electrochemical deposition, the PB layer was activated by CV in the potential range of −0.1 V–+0.4 V Ag/AgCl/KCl_3 mol L_^−1^ at a scan rate of 50 mV/s for 25 cycles. Then, the PB/AuNS/GR electrode was dried at 50 °C for about 15 min. and characterized by an FE-SEM [16,24].

### 2.3. Immobilization of GOx on a PB/AuNS/GR Electrode

A total of 3.0, 6.0 or 9.0 μL of 40 mg mL^−1^ GOx solution was deposited on the GR electrode surface, which was premodified with AuNS and PB (PB/AuNS/GR). After drying, the working electrode (GOx/PB/AuNS/GR) was coated with 3 µL of Nf (0.5%) (Nf- GOx/PB/AuNS/GR) at room temperature to prevent GOx from leaking off and to stabilize the formed structures on the electrode. After the addition of Nf layer, the electrodes were dried and then were washed with PBS solution before the electrochemical measurements were conducted.

### 2.4. Electrochemical Measurements

All electrochemical measurements were performed in PBS with 0.1 mol L^−1^ KCl using a three-electrode system. Platinum wire was used as an auxiliary electrode. Chronoamperometric measurements were performed at a constant −0.05 V potential vs. Ag/AgCl/KCl_3 mol L_^−1^. To begin with, a steady baseline current was attained, and then the PB/AuNS/GR electrode was tested by recording the currents after the addition of H_2_O_2_ in concentrations ranging from 0.05 to 11 mmol L^−1^. The same protocol was used for the biosensor based on the Nf-GOx/PB/AuNS/GR electrode using glucose concentrations ranging from 0.1 to 10 mmol L^−1^. The average results from at least three independent electrochemical measurements are presented.

The statistics software, SigmaPlot software (version 11.00), was used for the presentation of the experimental results and the determination of the slope, the correlation coefficient of the calibration curve, the difference in maximal current response registered during the enzymatic reaction (Δ*I_max_*) and the apparent Michaelis constant (*K*_M(app)_). Equation (2) was used to calculate the limit of detection (LOD).

(2) $$LOD=\frac{3\sigma }{S}$$

where *σ* is the standard deviation of the sample with the lowest glucose concentration response, and *S* is the slope of the calibration curve.

## 3. Results

**Figure 1:**
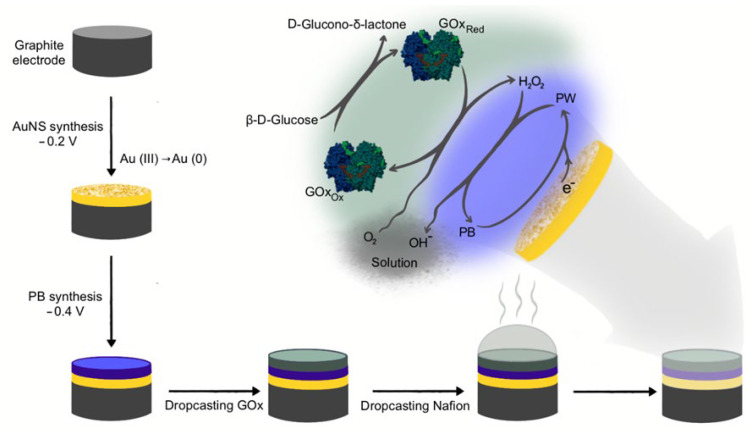
Schematic representation of the glucose biosensor preparation and glucose determination. The electrochemical synthesis of AuNS and PB on the GR electrode was performed, followed by GOx immobilization and stabilization by Nf.

In this research, a novel reagentless Nf-GOx/PB/AuNS nanobiocomposite-based amperometric biosensor for glucose determination was designed (Figure 1). In the previous research, dendritic gold nanostructures were successfully applied in the design of glucose biosensors using water-soluble (phenazine methosulfate) [1,3,6] and insoluble redox mediators (ferrocenecarboxylic acid, 1,10-phenathroline-5,6-dione and tetrathiafulvalene [3]). However, the concentration of glucose in the sample can be evaluated via the detection of the **H_2_O_2_** produced during the enzymatic oxidation of glucose in the presence of oxygen (Equations (3) and (4)):

(3) $$\mathrm{GOx}(\mathrm{FAD})+\beta \text{-}D\text{-}\mathrm{glucoseg}\overset{}{\to }\mathrm{GOx}({\mathrm{FADH}}_{2})+\beta \text{-}D\text{-}\mathrm{gluconolactone}$$

(4) $$\mathrm{GOx}({\mathrm{FADH}}_{2})+O_{2}\overset{}{\to }\mathrm{GOx}(\mathrm{FAD})+H_{2}O_{2}$$

In this research, the reagentless glucose biosensor based on AuNPs and **PB** was developed. A more detailed explanation of all the processes is presented using equations and Figure 1. Firstly, **PB**, synthesized on the AuNS/GR electrode, is reduced electrochemically to the colorless form of Prussian white (**PW**) (Equation (5)). Then, **PW**, acting as an electron-transfer mediator, participates in the electrochemical reduction of **H_2_O_2_** [25,26], and during the same reaction, **PW** is reoxidized to **PB** (Equation (6)):

(5) $$PB+e^{-}\overset{}{\to }PW$$

(6) $$K_{2}{Fe}^{2+}{Fe}^{2+}(CN{)}_{6}(PW)-e^{-}\overset{H_{2}O_{2}}{\to }K{Fe}^{3+}{Fe}^{2+}(CN{)}_{6}(PB)+K^{+}$$

**Fe^2+^** and **Fe^3+^** in Equation (6) correspond to the different oxidation states of **Fe** atoms in the PB structure.

The PW-catalyzed electrochemical reduction of **H_2_O_2_** was registered at a lower potential if compared with the previously mentioned research, where various redox mediators were used. Therefore, the influence of interfering substances could be avoided, resulting in an improvement in the reliability of the glucose concentration measurement. In addition, the catalytic constant of **PB** is three orders of magnitude higher than that of natural peroxidases [27], allowing the development of highly sensitive biosensors. In the present study, we developed and evaluated biosensors based on the Nf-GOx/**PB**/AuNS/GR electrodes for the detection of glucose.

### 3.1. Selection of the Optimal Glucose Biosensor Performance Conditions

**Figure 2:**
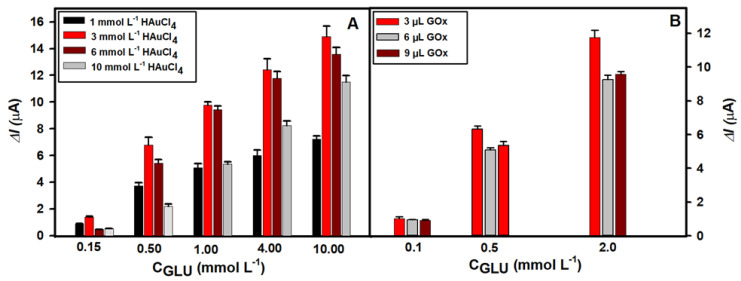
(**A**) Dependence of the current response of glucose biosensors based on Nf-GOx/PB/AuNS/GR electrodes, where AuNS were electrochemically deposited using 1.0, 3.0, 6.0 and 10.0 mmol L^−^^1^ concentrations of HAuCl solution containing 0.2 mol L^−^^1^ H_2_SO_4_ at a constant −0.2 V potential for 120 s. (**B**) Dependence of the current response of glucose biosensors based on AuNS electrodeposited using 3.0 mmol L^−^^1^ HAuCl_4_ concentration after deposition of different amounts of 40 mg/mL GOx.

The structure, size and distribution of AuNS on the surface of the GR electrode depend on a few factors. The concentration of HAuCl_4_ in the solution, the selected supporting electrolyte, the parameters of electrochemical synthesis and the duration of this process greatly impact the final morphology of AuNS [1,28]. In this study, H2SO4 was used as a supporting electrolyte in order to obtain AuNS of equal size which are uniformly distributed on the surface of GR electrode [23]. To select the optimal concentration of HAuCl_4_ and to develop a sensitive glucose biosensor, four HAuCl_4_ concentrations (1.0, 3.0, 6.0 and 10.0 mmol L^−^^1^) were used for the AuNS synthesis in the same conditions. After PB electrodeposition, GOx was then immobilized on the PB/AuNS nanocomposite-premodified electrodes and covered by Nf. Then, amperometric measurements were performed at −0.05 V in the 0.05 mol L^−^^1^ PBS solution with 0.1 mol L^−^^1^ KCl in the presence of different concentrations of glucose (from 0.1 to 10.0 mmol L^−^^1^). As evident in Figure 2, the modification of the GR electrodes with AuNS has a positive impact on the registered analytical signal in all cases. The highest peak current responses at different glucose concentrations were recorded using the Nf-GOx/PB/AuNS/GR electrode premodified with the AuNS obtained from a 3.0 mmol L^−^^1^ HAuCl_4_ solution when 3 µL of the GOx solution was deposited. The Δ*I_max_* at 0.5 mmol L^−^^1^ of glucose was 6.70 ± 0.10 µA. However, the Δ*I_max_* obtained with the electrodes premodifed with AuNS using 1.0, 6.0 and 10.0 mmol L^−^^1^ of HAuCl_4_ was 1.8, 1.2 and 3.1 times lower, respectively. The same tendency was observed at higher glucose concentrations. Namely, 2.1, 1.1 and 1.29 times lower Δ*I_max_* were obtained at the 10.0 mmol L^−^^1^ of glucose. Based on the obtained results, a 3.0 mmol L^−^^1^ HAuCl_4_ concentration was chosen as the optimal one for the AuNS synthesis and Nf-GOx/PB/AuNS/GR electrode-based glucose biosensor development.

The signal response of the biosensor is highly dependent on the amount of immobilized enzyme. In the next step of biosensor optimization, the optimal amount of GOx on the PB/AuNS/GR electrode was selected. AuNS were synthesized from the 3.0 mmol L^−1^ HAuCl_4_ aqueous solution. Then, 3.0, 6.0 or 9.0 µL of the 40 mg/mL concentration GOx solution was deposited. The amperometric responses to the different glucose concentrations were recorded in the 0.05 mol L^−1^ PBS solution by applying a −0.05 V potential to the electrode. The data presented in Figure 2B show that the maximal signal response at 0.5 mmol L^−1^ of glucose was registered after the deposition of 3 µL of GOx solution (Δ*I_max_* = 6.32 ± 0.03 µA). Using 6 and 9 µL of GOx solution, the registered analytical signals were slightly lower (Δ*I_max_* = 5.08 ± 0.03 µA and 5.36 ± 0.04 µA). The decrease in the analytical signal might be mainly due to the substrate and enzymatic reaction product diffusion limitations at the thicker enzyme layer formation. Therefore, 3.0 µL of GOx on the PB/AuNS/GR electrode surface was considered to be the optimal amount for the development of a glucose biosensor.

Cyclic voltammograms were registered at each step of the electrode modification in the −0.05–+0.4 V range vs. AgCl/KCl_3 mol L_^−1^ at the potential scan rate of 50 mV/s. Figure S1 illustrates that there are no oxidation and reduction current peaks in the cyclic voltammograms recorded using the GR and AuNS/GR electrodes. However, the increased area of the voltammogram after the electrochemical deposition of AuNS due to an increase in the electrochemical capacity confirms the successful modification of the electrode. Meanwhile, the oxidation and reduction current peaks appeared after the modification of the electrodes with PB due to the electrochemical reaction of high-spin ferric ions in PB (Fe^2+^/Fe^3+^ transition). The well-visible oxidation and reduction peaks appeared in the cyclic voltammograms using Nf-GOx/PB/AuNS/GR in a wider potential scan range (−0.2–+0.7 V). In addition, the increase in the Nf-GOx/PB/AuNS/GR reduction peak after the addition of 1 mmol L^−1^ of glucose indicates that the H_2_O_2_ produced during the enzymatic reaction was successfully electrochemically reduced at the electrode surface (Figure S2).

### 3.2. Characterization of AuNS and PB/AuNS Nanocomposite Electrochemically Deposited on GR Electrode

**Figure 3:**
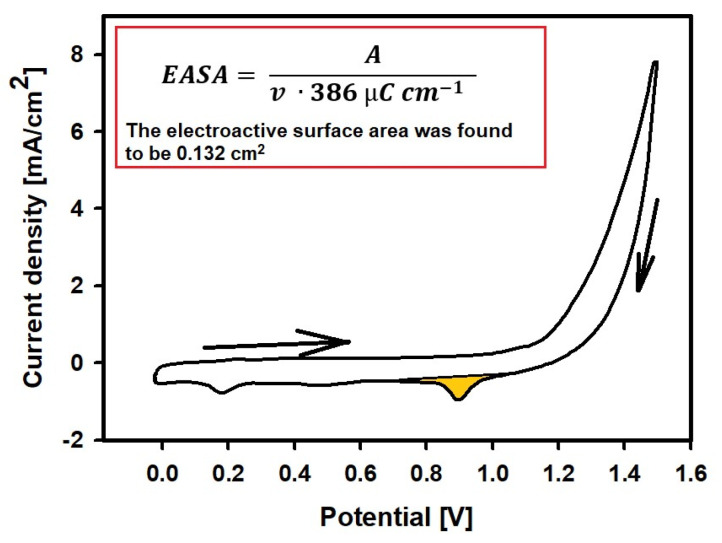
Cyclic voltammogram of AuNS/GR electrode recorded in 0.5 mol L^−1^ H_2_SO_4_ solution at the 100 mV/s potential sweep rate.

After choosing the optimal conditions for the AuNS synthesis on the GR electrode surface, the electrochemically active surface area of the AuNS was evaluated by the CV in a 0.5 mol L^−1^ H_2_SO_4_ solution at the 100 mV/s potential sweep rate [1,23]. During this measurement, both the electrochemical oxidation of gold and subsequent reduction occurs (Figure 3). The electroactive surface area was found to be 0.132 cm^2^ for the electrochemically deposited AuNS using the 3.0 mmol L^−1^ concentration of HAuCl_4_ solution in the presence of 0.2 mol L^−1^ H_2_SO_4_. Thus, the electrochemically active surface area was enhanced by 1.86 times, as the AuNS was successfully formed on the GR electrode surface.

**Figure 4:**
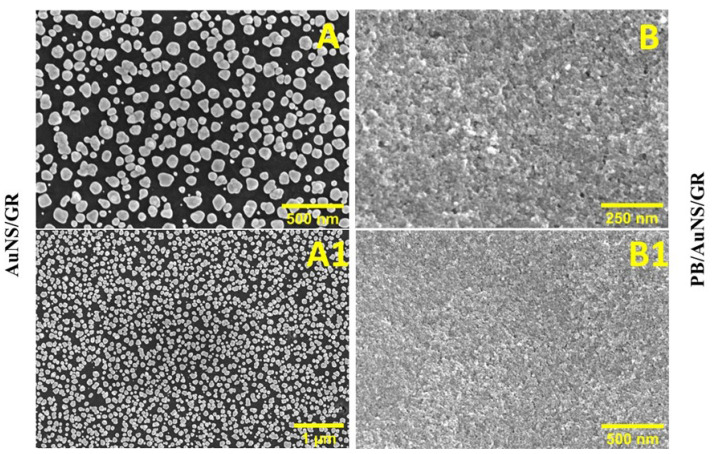
FE-SEM images of AuNS (**A**,**A1**) and PB/AuNS nanocomposite (**B**,**B1**) electrodeposited on the GR electrode.

GR electrodes modified with AuNS and with PB/AuNS nanocomposite in the best biosensor performance conditions, were examined by FE-SEM. AuNS are polydisperse and mostly circular, with an average diameter of 84 ± 35 nm; however, not the whole GR electrode surface is covered by AuNS (Figure 4A,A1). The surface of the electrode after the PB/AuNS nanocomposite deposition is smoother, and almost all of the GR electrode surface is occupied by the nanocomposite. The obtained results confirm the successful synthesis of AuNS and PB and allow us to conclude that PB was electrodeposited on AuNS and the uncovered GR electrode surface (Figure 4B,B1).

### 3.3. Characterization of the Developed Glucose Biosensor Based on Nf-GOx/PB/AuNS/GR Electrode

**Figure 5:**
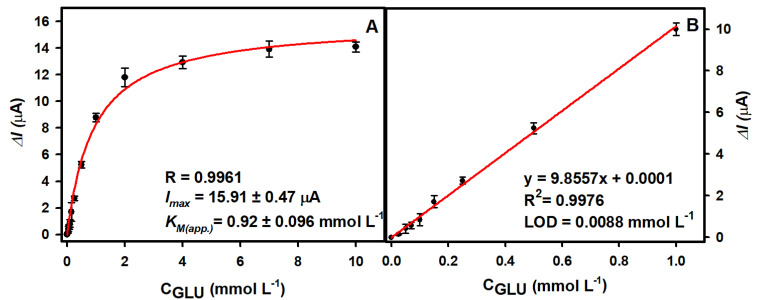
(**A**) Calibration plot of glucose biosensor based on Nf-GOx/PB/AuNS/GR electrochemically deposited on the electrode in optimal conditions. (**B**) The linear range of glucose biosensors.

The performance of the glucose biosensor, developed in optimal conditions, was investigated (Figure 5). The registered current response followed a hyperbolic relationship in the glucose concentration range from 0.025 to 10.0 mmol L^−1^. The Δ*I_max_* of the developed biosensor was 15.91 ± 0.47 µA (*R* = 0.9961), while the *K_M_* was 0.92 ± 0.096 mmol L^−1^. The linear range (LR) was obtained in the range from 0.025 to 1 mmol L−1 (R2=0.9976) with a LOD of 0.0088 mmol L−1 (S/N = 3). The dynamic range was significantly wider and covered all tested glucose concentrations from 0.025 to 10.0 mmol L^−1^.

The analytical parameters of the glucose biosensors based on PB are compared in Table 1. As evident from the summarized results, the developed biosensor exhibited 5.7 times lower LOD if compared with a glucose biosensor based on a carbon cloth electrode modified with coral-like gold micro/nanostructures and PB (GOx@PB/coral-like AuNS/CC) [16], was more than 11 times lower if compared with a biosensor based on nitrogen-doped graphite foam electrode with PB particles (GOx/PB/NGF) [29] and was about 17 times lower if compared with carbon screen-printed electrodes based on porous graphene aerogel and PB and covered by chitozan (CTS/GOx/GA@PB/carbon SPE) [30]. It shows a lower LOD in almost all discussed biosensors except the biosensor based on PB-gold nanocomposite films and platinum nanoclusters deposited on the GC electrode (Nf- GOx/Pt-NCs/PB-Au/GC) [4]. However, in the mentioned biosensor, a more intricate electrode modification procedure was employed, involving the combination of PB–gold nanocomposite films and platinum nanoclusters. Despite the mentioned advantages, this biosensor is based on a GC electrode applying a few nanostructures, and the preparation of such biosensors is more complicated. The LR of the developed biosensor is in the range of 0.025–1.0 mmol L^−1^ of glucose; thus, it can operate in the lower glucose concentrations in comparison to biosensors based on a GOx@PB/coral-like AuNS/CC electrode [16]; however, the upper range is lower and similar to the biosensor based on the Nf-GOx/Pt-NCs/PB-Au/GC electrode [4]. The batch-to-batch variation of the presented biosensors, evaluated based on the maximal signal response, showed a low relative standard deviation of 1.69%. Additionally, the developed novel amperometric glucose biosensor can operate well when detecting glucose in the diluted samples, ensuring the applicability of the biosensor to detect glucose in the clinically important glucose concentration range.

### 3.4. Study of Analytical Signal Repeatability and Influence of Interfering Substances on the Analytical Signal

**Figure 6:**
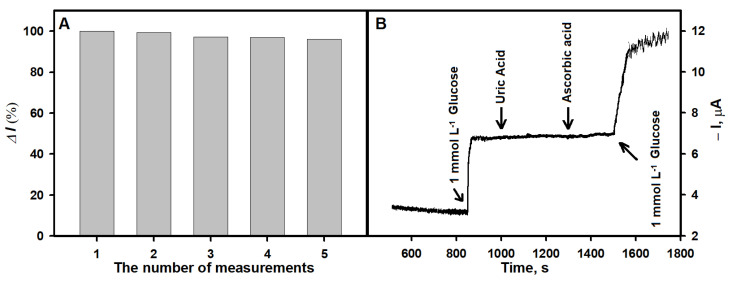
(**A**) The response of the biosensor based on the Nf-GOx/PB/AuNS/GR electrode during repeated detection of glucose (1 mmol L^−1^). (**B**) The effect of interfering substances on the amperometric response (0.1 mmol L^−1^ UA, 0.1 mmol L^−1^ AA).

The repeatability of the analytical signal response and the influence of interfering electrochemically active substances on the registered signal are important characteristics of a biosensor. In the study focused on repeatability, 1 mmol L^−1^ of glucose concentration was selected for the current response measurements. The current response decreased by 0.7% after the second measurement. After five measurements, 96% of the initial current response was retained (Figure 6A). To evaluate the influence of interfering species on the analytical signal, the current response of the biosensor was measured with the addition of 1.0 mmol L^−1^ glucose and 0.1 mmol L^−1^ of electrochemical-interfering substances, uric acid (UA) and ascorbic acid (AA). As shown in Figure 6B, the biosensor responded rapidly to the addition of 1.0 mmol L^−1^ glucose, while the negligible current response to the addition of 0.1 mmol L^−1^ UA and 0.1 mmol L^−1^ AA was observed. When 1.0 mmol L^−1^ glucose was added to the solution containing the tested interfering substances, the response of the biosensor exhibited the same behavior as after the addition of glucose the first time. These exceptional results can be elucidated by the use of low applied potential due to the introduction of PB into the biosensor system [33,34] and Nf film at the sensing interface that forms a network structure, which further inhibits electroactive interferents from penetrating the film [35].

Moreover, the study assessed the influence of structurally similar monosaccharides to glucose, such as xylose, mannose, fructose and galactose, on the amperometric response. The results presented in Figure S3 showed no response upon the addition of 1 mmol L^−1^ xylose, mannose, fructose or galactose to the electrochemical cell filled with 0.05 mol L^−1^ PBS with 0.1 mol L^−1^ KCl (pH 5.8). Consequently, under the specified experimental conditions, the developed biosensor demonstrated selectivity specifically towards glucose.

### 3.5. Determination of H_2_O_2_ Using Biosensor Based on PB/AuNS/GR Electrode

**Figure 7:**
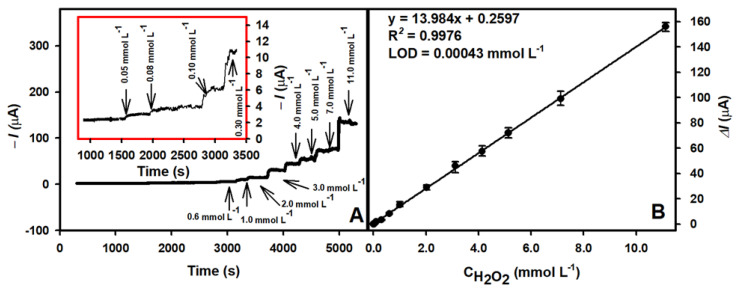
(**A**) The current response of the biosensor based on the PB/AuNS/GR electrode after the addition of H_2_O_2_. (**B**) The calibration plot of the amperometric response vs. H_2_O_2_ concentration. The amperometric response at −0.05 V was registered in 0.05 M PBS solution with 0.1 M KCl.

To confirm the ability of the biosensor to determine H_2_O_2_, the PB/AuNS/GR electrode was exposed to different concentrations of H_2_O_2_. Figure 7A shows the typical current–time curve registered in PBS solution containing 0.1 mol L^−1^ KCl at the applied −0.05 V potential after the addition of H_2_O_2_. The linear dependence of (Figure 7B) along with the increased H_2_O_2_ concentration was observed in the range of 0.05–11.0 mmol L^−1^. The LOD for H_2_O_2_ was calculated as 0.00043 mmol L^−1^.

### 3.6. Application of Developed Biosensor for Glucose Determination in Human Serum Sample

After evaluating the influence of interfering substances, the performance of the developed biosensor was tested in the human serum 10 times diluted with 0.05 mol L^−1^ PBS solution with 0.1 mol L^−1^ KCl. The serum before the analysis was prepared according to the earlier described procedure [7]. The 0.49 mmol L^−1^ concentration of glucose was determined by a commercial glucometer in the diluted serum sample. Electrochemical measurements were performed after the addition of a known concentration of glucose. Measurements at each concentration of glucose were repeated three times, and the averages of the obtained responses are presented in Table 2. The recovery of glucose in human serum was in the range of 101–102%. Therefore, the developed glucose biosensor based on the Nf-GOx/PB/AuNS/GR electrode can be successfully applied for glucose determination in real samples with sufficient accuracy.

## 4. Conclusions

In this study, a novel and simplified in-operation first-generation amperometric biosensor for glucose determination was developed by modifying GR electrodes with electrochemically deposited AuNS and PB and immobilized GOx with the assistance of Nf. The electrochemical biosensor based on the Nf-GOx/PB/AuNS/GR electrode was simple to operate and exhibited good repeatability, and the LOD and LR were suitable for the determination of low glucose concentrations with high resistance to other electroactive substances present in the real samples. Additionally, it was shown that the proposed GR electrode modification with the AuNS and PB procedure is suitable for the development of a highly sensitive H_2_O_2_ sensor. The obtained results show a very promising direction for the improvement in the amperometric biosensor’s performance. Increase in electroactive surface area and improvement in GOx immobilization efficiency can be achieved by modifying the electrode surface with AuNS. In addition, the use of PB can facilitate electrochemical reduction of the H_2_O_2_ produced during enzymatic oxidation of glucose at low potentials. The developed reagentless biosensor is suitable for the analysis of diluted real samples and the monitoring of glucose levels with high precision and accuracy.

## Figures and Tables

**Table 1:** Comparison of glucose biosensors based on PB.

Modified Working Electrode	Potential/Solution	LOD	LR	Ref.
Nf-GOx/Pt-NCs/PB-Au/GC	−0.15 V/0.1 mol L^−1^ KNO_3_ (pH 6.0)	0.001 mmol L^−1^	0.003–1.1 mmol L^−1^	[4]
CTS/GOx/GA@PB/carbon SPE	−0.35 V/0.01 mol L^−1^ PBS	0.15 mmol L^−1^	0.5–6.0 mmol·L^−1^	[30]
GOx@PB/coral-likeAuNS/CC	−0.05 V/0.1 mol L^−1^ PBS with 0.1 mol L^−1^ KCl (pH 6.0)	0.05 mmol L^−1^	0.15–6.65 mmol·L^−1^	[16]
GOx-PCPB/Pt	−0.05 V/0.1 mol L^−1^ PBS with 0.1 mol L^−1^ KCl (pH 6.5)	0.03 mmol L^−1^	0.03–0.4 mmol·L^−1^	[31]
GOx/PB/NGF	−0.05 V/0.1 mol L^−1^ PBS with 0.1 M KCl (pH 6.0)	0.1 mmol L^−1^	0.2–20.0 mmol L^−1^	[29]
GOx–PDA/PB/GC	−0.0 V/0.05 mol L^−1^ PBS with 0.1 mol L^−1^ KCl (pH 7.4).	0.0462 mmol L^−1^	0.2–3.4 mmol L^−1^	[32]
Nf/GOx/PB/AuNS/GR	−0.05 V/0.05 mol L^−1^ PBS with 0.1 mol L^−1^ KCl (pH 5.8).	0.0088 mmol L^−1^	0.025–1.0 mmol L^−1^	This work

Abbreviations: Nf—Nafion; GOx—glucose oxidase; PB—Prussian blue; Pt-NCs—platinum nanoclusters; PB–Au—Prussian blue–gold nanocomposite; CTS—chitosan; GA@PB—graphene aerogel and Prussian blue composite material; GC—glassy carbon electrode; SPE—screen-printed electrode; CC—carbon cloth; PCPB—porous carbon Prussian blue; Pt—platinum electrode; NGF—nitrogen-doped graphite foam electrode; PB/NGF-NGF with Prussian blue particles; PDA—self-polymerized dopamine; AuNS—gold nanostructures; GR—graphite rod electrode.

**Table 2:** Recovery of glucose in human serum using an electrochemical biosensor based on Nf-GOx/PB/AuNS/GR electrode **.

Concentration in the Serum, mmol L^−1^ *	Added Concentration, mmol L^−1^	Detected Concentration, mmol L^−1^	Recovery, %	RSD, % (n = 3)
0.49	0.5	1.01	102	0.84
0.49	1.0	1.51	101	0.74
0.49	1.5	2.02	101	0.69

* The concentration of glucose in the serum was determined with a glucometer FreeStyle Optium ART16648 Rev. B 05/10. ** Amperometric response was measured in the human serum diluted 10 times using 0.05 mol L^−1^ PBS solution with 0.1 mol L^−1^ KCl.

## Data Availability

The data presented in this study are available on request from the first author.

## Supplementary Materials

The following supporting information can be downloaded at: https://www.mdpi.com/article/10.3390/bios13100942/s1, Figure S1: Cyclic voltammograms of bare GR, AuNS/GR, PB/AuNS/GR, Nf-GOx/PB/AuNS/GR electrodes in 0.05 mol L^−1^ PBS with 0.1 mol L^−1^ KCl (pH 5.8) at the potential scan rate of 50 mV s^−1^; Figure S2: Cyclic voltammograms of the Nf-GOx/PB/AuNS/GR in 0.05 mol L^−1^ PBS with 0.1 mol L^−1^ KCl (pH 5.8) solution containing 0.1 M KCl; Figure S3: The amperometric responce of the biosensor based on Nf-GOx/PB/AuNS/GR electrode after the addition of 1 mmol L^−1^ glucose, 1 mmol L^−1^ xylose, 1 mmol L^−1^ mannose, 1 mmol L^−1^ fructose, 1 mmol L^−1^ galactose and 1 mmol L ^−1^ glucose in 0.05 mol L^−1^ PBS with 0.1 mol L^−1^ KCl (pH 5.8).
