# Supplementary material for: The Development of Reagentless Amperometric Glucose Biosensor Based on Gold Nanostructures, Prussian Blue and Glucose Oxidase
Source: Biosensors (Basel). 2023 Oct 20;13(10):942. doi: 10.3390/bios13100942 (PMC10605372; doi:10.3390/bios13100942)
Supplement: Supplementary file 1 [file biosensors-13-00942-s001.zip › biosensors-2604816-supplementary.pdf]

# The Development of Reagentless Amperometric Glucose Biosensor Based on Gold Nanostructures, Prussian Blue and Glucose Oxidase

**Laura Sakalauskiene [1], Benediktas Brasiunas [1], Anton Popov [1,2], Asta Kausaite-Minkstimiene [1,2,*] and Almira Ramanaviciene [1,2,*]**

[1] NanoTechnas—Center of Nanotechnology and Materials Science, Faculty of Chemistry and Geosciences, Vilnius University, Naugarduko St. 24, LT-03225 Vilnius, Lithuania; laura.sakalauskiene@chgf.vu.lt (L.S.); benediktas.brasiunas@chgf.vu.lt (B.B.); anton.popov@chgf.vu.lt (A.P.)

[2] Department of Immunology, State Research Institute Centre for Innovative Medicine, Santariskiu St. 5, LT-08406 Vilnius, Lithuania

* Correspondence: asta.kausaite@chf.vu.lt (A.K.-M.); almira.ramanaviciene@chf.vu.lt (A.R.)

## Supplementary data

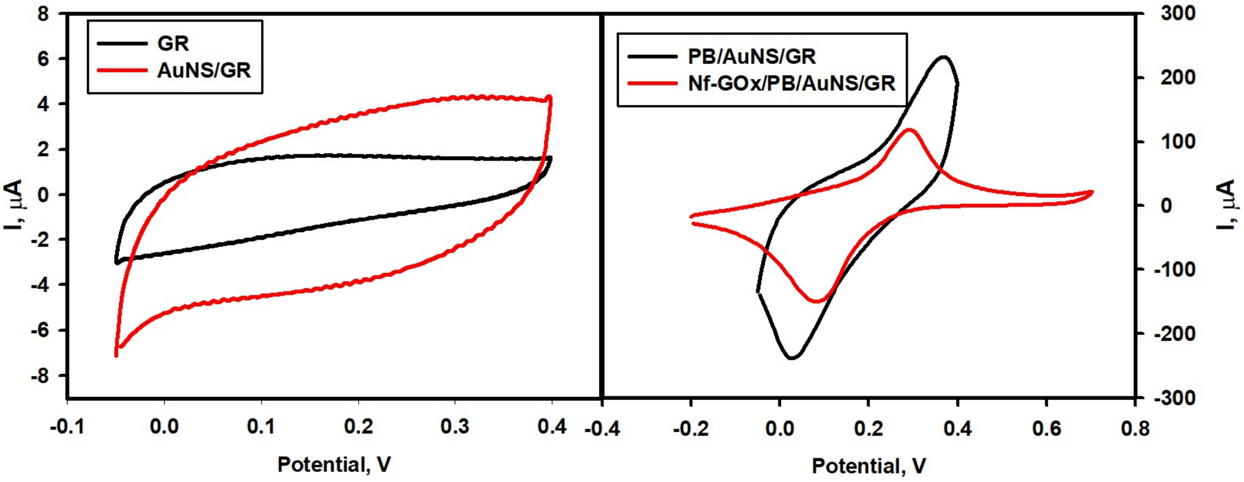


**Fig. S1.** Cyclic voltammograms of bare GR, AuNS/GR, PB/AuNS/GR, Nf-GOx/PB/AuNS/GR electrodes in 0.05 mol $L^{-1}$ PBS with 0.1 mol $L^{-1}$ KCl (pH 5.8) at the potential scan rate of 50 mV/s.

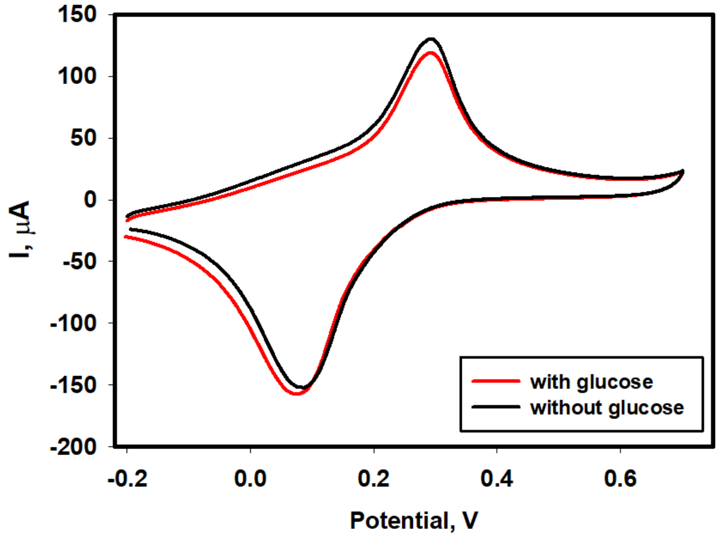


**Fig. S2.** Cyclic voltammograms of the Nf-GOx/PB/AuNS/GR in 0.05 mol $L^{-1}$ PBS with 0.1 mol $L^{-1}$ KCl (pH 5.8) solution containing 0.1 M KCl: without (black line) and with 1 mmol $L^{-1}$ glucose (red line) at the potential scan rate of 50 mV/s.

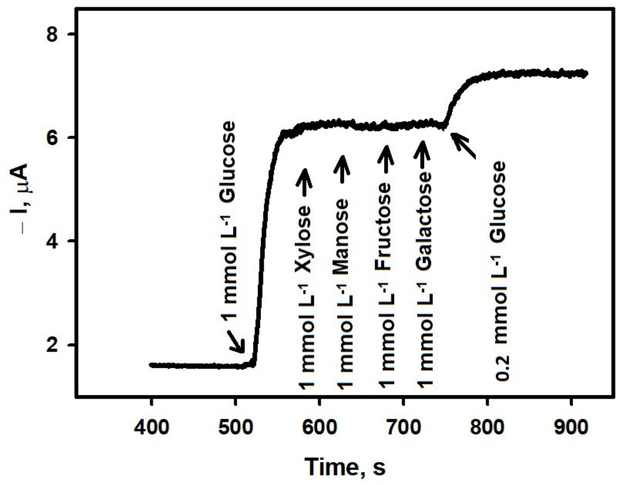


**Fig. S3.** The amperometric response of the biosensor based on Nf-GOx/PB/AuNS/GR electrode after the addition of 1 mmol $L^{-1}$ glucose, 1 mmol $L^{-1}$ xylose, 1 mmol $L^{-1}$ mannose, 1 mmol $L^{-1}$ fructose, 1 mmol $L^{-1}$ galactose and 1 mmol $L^{-1}$ glucose in 0.05 mol $L^{-1}$ PBS with 0.1 mol $L^{-1}$ KCl (pH 5.8).
